# Analgosedation in patients with acute respiratory failure on noninvasive ventilation: is it truly safe?

**DOI:** 10.1186/s44158-024-00221-y

**Published:** 2024-12-21

**Authors:** Manuel Alberto Guerrero-Gutiérrez, Rafael Alfonso Reyes-Monge, Ignacio Rodríguez-Guevara, Diego Escarramán-Martínez, Orlando Rubén Pérez-Nieto

**Affiliations:** 1 Department of Anesthesiology and Intensive Care Unit, Baja Medical Center, Tijuana, Baja California, Mexico; 2 Intensive Care Unit, Hospital General San Juan del Rio, Queretaro, Mexico; 3 Department of Anesthesiology, Centro Medico Nacional “La Raza” IMSS, Mexico City, Mexico

Dear Editor,

We read with interest the Delphi consensus on the management of analgosedation during noninvasive respiratory support [1] and believe that it is important to comment on the following considerations regarding sedation in patients receiving noninvasive ventilation (NIV) for acute respiratory failure (ARF).

Notably, the incidence of bradycardia is greater when dexmedetomidine is used during NIV (*N* = 565; *RR* 1.90; 95% *CI* 1.03–3.49; *P* = 0.04; *I*^2^ = 0%) [2]. For this reason, the Delphi consensus authors recommend continuous monitoring of vital signs—a practice that, in resource-limited settings with minimal clinical staff and no telemetry, may carry significant risks with this drug. Additionally, other adverse effects that could compromise ventilation, such as vomiting, gastric aspiration, respiratory tract infection, and respiratory depression, have not been thoroughly evaluated [2].

The study by Yang et al. provides evidence on the potential benefits of sedation in reducing the need for intubation and the risk of delirium, as well as on the advantage of dexmedetomidine over other sedatives, albeit with a higher incidence of bradycardia [2]. This additional discussion enriches our evaluation of the risks and benefits of sedation in NIV.

A recent large retrospective cohort study [3] involving patients with ARF due to obstructive lung disease, acute respiratory distress syndrome (ARDS), congestive heart failure, and mixed respiratory failure who received NIV (*n* = 433,357) revealed that 26.7% (95% *CI*, 26.3–27.0%) of these patients received sedation or analgesia. Among these patients, 50,589 (11.7%) received only opioids, 40,645 (9.4%) received only benzodiazepines, and 20,146 (4.6%) received both opioids and benzodiazepines. Additionally, 1573 patients (0.4%) were administered dexmedetomidine alone, and 2639 (0.6%) received dexmedetomidine in addition to opioids and/or benzodiazepines.

The risks of potential harm in NIV vary depending on the type of sedative or analgesic used (e.g. benzodiazepines) and the patient’s clinical characteristics. Certain medications, such as benzodiazepines and opioids, are associated with a higher risk of NIV failure and mortality in patients with specific conditions, such as those at risk of developing ARDS or congestive heart failure.

The use of sedation was associated with increased odds of requiring intubation or mortality: opioids alone (*OR* 1.23, 95% *CI* 1.20–1.27), benzodiazepines alone (*OR* 1.46, 95% *CI* 1.41–1.51), opioids and benzodiazepines together (*OR* 1.61, 95% *CI* 1.55–1.68), dexmedetomidine alone (*OR* 1.96, 95% *CI* 1.71–2.24), and dexmedetomidine combined with opioids and/or benzodiazepines (*OR* 1.91, 95% *CI* 1.72–2.13), all with *p* < 0.001. These results were consistent across multivariable models, which included available confounders, as well as an inverse probability weighting model. Despite the limitations of this study due to its observational nature, these findings suggest that the use of sedation and/or analgesia during NIV for ARF in patients with chronic obstructive pulmonary disease (COPD) and those at risk of acute respiratory distress syndrome (ARDS) (*OR* 1.54, 95% *CI* 1.49–1.58), congestive heart failure (*OR* 0.56, 95% *CI* 0.53–0.58), or mixed respiratory failure (OR 0.86, 95% *CI* 0.84–0.89) may carry potential harm by increasing both mortality risk and the likelihood of death and especially in those at risk of ARDS.

Paradoxically, despite real-world data indicating that the use of opioids during NIV for ARF is associated with a lower risk of intubation and death, their use has been declining in the USA over the past 10 years. Moreover, the use of dexmedetomidine has been increasing [2], despite safety alerts in Europe and Australia due to the increased mortality observed in intubated patients younger than 63.7 years compared with other sedatives [4].

In conclusion, these observations highlight the importance of critically evaluating sedation practices in patients with acute respiratory failure undergoing noninvasive ventilation. Further research is needed to ensure that sedative protocols align with patient safety and outcomes, particularly given the complex and potentially adverse effects associated with these interventions.

## Data Availability

No datasets were generated or analysed during the current study.

## Abbreviations

NIV: Noninvasive ventilation
ARDS: Acute respiratory distress syndrome
ARF: Acute respiratory failure
RR: Relative risk
CI: Confidence interval
OR: Odds ratio
